# ACE2-IgG1 fusions with improved *in vitro* and *in vivo* activity against SARS-CoV-2

**DOI:** 10.1016/j.isci.2021.103670

**Published:** 2021-12-20

**Authors:** Naoki Iwanaga, Laura Cooper, Lijun Rong, Nicholas J. Maness, Brandon Beddingfield, Zhongnan Qin, Jackelyn Crabtree, Ralph A. Tripp, Haoran Yang, Robert Blair, Sonia Jangra, Adolfo García-Sastre, Michael Schotsaert, Sruti Chandra, James E. Robinson, Akhilesh Srivastava, Felix Rabito, Xuebin Qin, Jay K. Kolls

**Affiliations:** 1 Departments of Pediatrics and Medicine, Center for Translational Research in Infection and Inflammation, Tulane University School of Medicine, New Orleans, LA 70112, USA; 2 Departments of Microbiology and Immunology, College of Medicine University of Illinois at Chicago, Chicago, IL 60612, USA; 3 Departments of Microbiology and Immunology, Tulane University School of Medicine, New Orleans, LA 70112, USA; 4 Departments of Infectious Diseases, Animal Health Research Center, University of Georgia, Athens, GA 30602,USA; 5 Departments of Pathology and Laboratory Medicine, Tulane University School of Medicine, New Orleans, LA 70112, USA; 6 Tulane National Primate Research Center, Covington, LA 70433, USA; 7 Department of Microbiology, Icahn School of Medicine at Mount Sinai, New York, NY 10029, USA; 8 Global Health and Emerging Pathogens Institute, Icahn School of Medicine at Mount Sinai, New York, NY 10029, USA; 9 Department of Medicine, Division of Infectious Diseases, Icahn School of Medicine at Mount Sinai, New York, NY 10029, USA; 10 The Tisch Cancer Institute, Icahn School of Medicine at Mount Sinai, New York, NY 10029, USA; 11 Department of Department of Pathology, Molecular and Cell-Based Medicine, Icahn School of Medicine at Mount Sinai, New York, NY 10029, USA; 12 Departments of Pediatrics, Tulane University School of Medicine, New Orleans, LA 70112, USA

**Keywords:** Drugs, Virology

## Abstract

SARS-CoV-2, the etiologic agent of COVID-19, uses ACE2 as a cell entry receptor. Soluble ACE2 has been shown to have neutralizing antiviral activity but has a short half-life and no active transport mechanism from the circulation into the alveolar spaces of the lung. To overcome this, we constructed an ACE2-human IgG1 fusion protein with mutations in the catalytic domain of ACE2. A mutation in the catalytic domain of ACE2, MDR504, significantly increased binding to SARS-CoV-2 spike protein, as well as to a spike variant, *in vitro* with more potent viral neutralization in plaque assays. Parental administration of the protein showed stable serum concentrations with excellent bioavailability in the epithelial lining fluid of the lung, and ameliorated lung SARS-CoV-2 infection *in vivo*. These data support that the MDR504 hACE2-Fc is an excellent candidate for treatment or prophylaxis of COVID-19 and potentially emerging variants.

## Highlights

• Several mutations in the ACE2 catalytic domain retain SARS-Cov-2 binding
• An H345A mutation showed enhanced spike binding/neutralization
• H345A ACE2-IgG1 fusion showed efficacy in preclinical SARS-CoV-2 infection

## Graphical abstract

**Figure:**
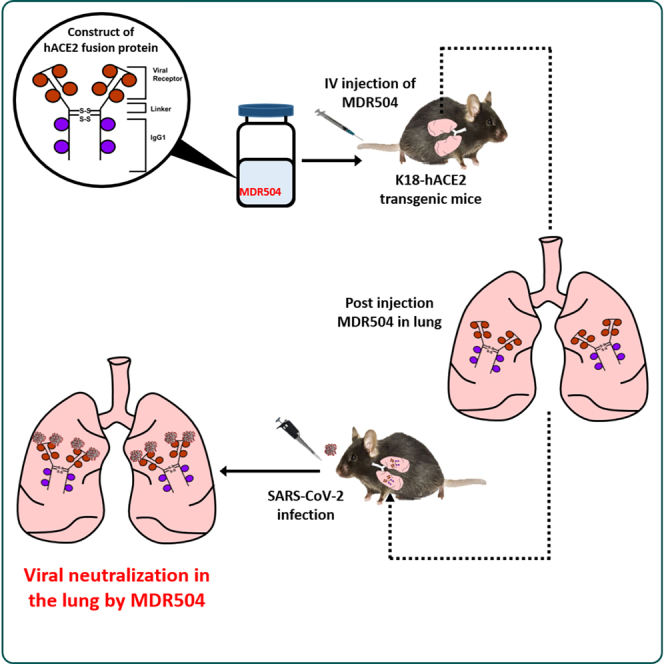

## Introduction

SARS-CoV-2, the etiologic agent of COVID-19, uses ACE2 as a cell entry receptor (Xu et al., 2020). Soluble ACE2 has been shown to have neutralizing antiviral activity but has a short half-life and no active transport mechanism from the circulation into the alveolar spaces of the lung (Lei et al., 2020). To overcome this, we constructed an ACE2-human IgG1 fusion protein with mutations in the catalytic domain of ACE2. MDR503 has a R273A mutation, MDR504 has a H345A mutation, and MDR505 has the R273A/H345A mutation. This fusion protein contained a LALA mutation that abrogates Fcrγ binding but retains FcRN binding. A mutation in the catalytic domain of ACE2, MDR504, had significantly increased binding to SARS-CoV-2 spike protein, as well as to several spike variants, *in vitro* with more potent viral neutralization in a plaque assay. These data were independently validated by the Coronavirus Immunotherapy Consortium (CoVIC). Parental administration of the MDR504 protein showed stable serum concentrations with excellent bioavailability in the epithelial lining fluid of the lung, and ameliorated lung SARS-CoV-2 infection *in vivo*. Moreover, MDR504 retained binding to several emerging SARS-CoV-2 variants. These data support that the MDR504 hACE2-Fc is an excellent candidate for treatment or prophylaxis of COVID-19.

## Results

### *In vitro* activity of ACE2:Human IgG1 fusion proteins

In addition to the wild-type (WT) ACE2 ectodomain, we engineered several mutations in the catalytic domain and named these MDR503, MDR504, and MDR505. Constructs included an IgGκ leader sequence and an IEGR linker between ACE2 and the CH2 and CH3 domains of human IgG1 (Figure S1A). In addition, schematic overviews of the hACE2-Fc and MDR504 are depicted by Raptor X (Figure S1B) (Xu et al., 2008).

All constructs of hACE2-Fc expressed proteins consistent with homodimers after transfection in HEK293T cells. After transient transfection of the constructs in 293T cells, human IgG-Fc in the supernatant was readily detected by ELISA (data not shown). Proteins were run on a reduced SDS-PAGE gel and migrated at ∼ 140 kDa consistent with the predicted molecular weight of the monomer (Figure S1C). In addition, SDS-PAGE analysis in non-reducing conditions revealed migration consistent with a dimeric protein (Figure S1D).

**Figure 1:**
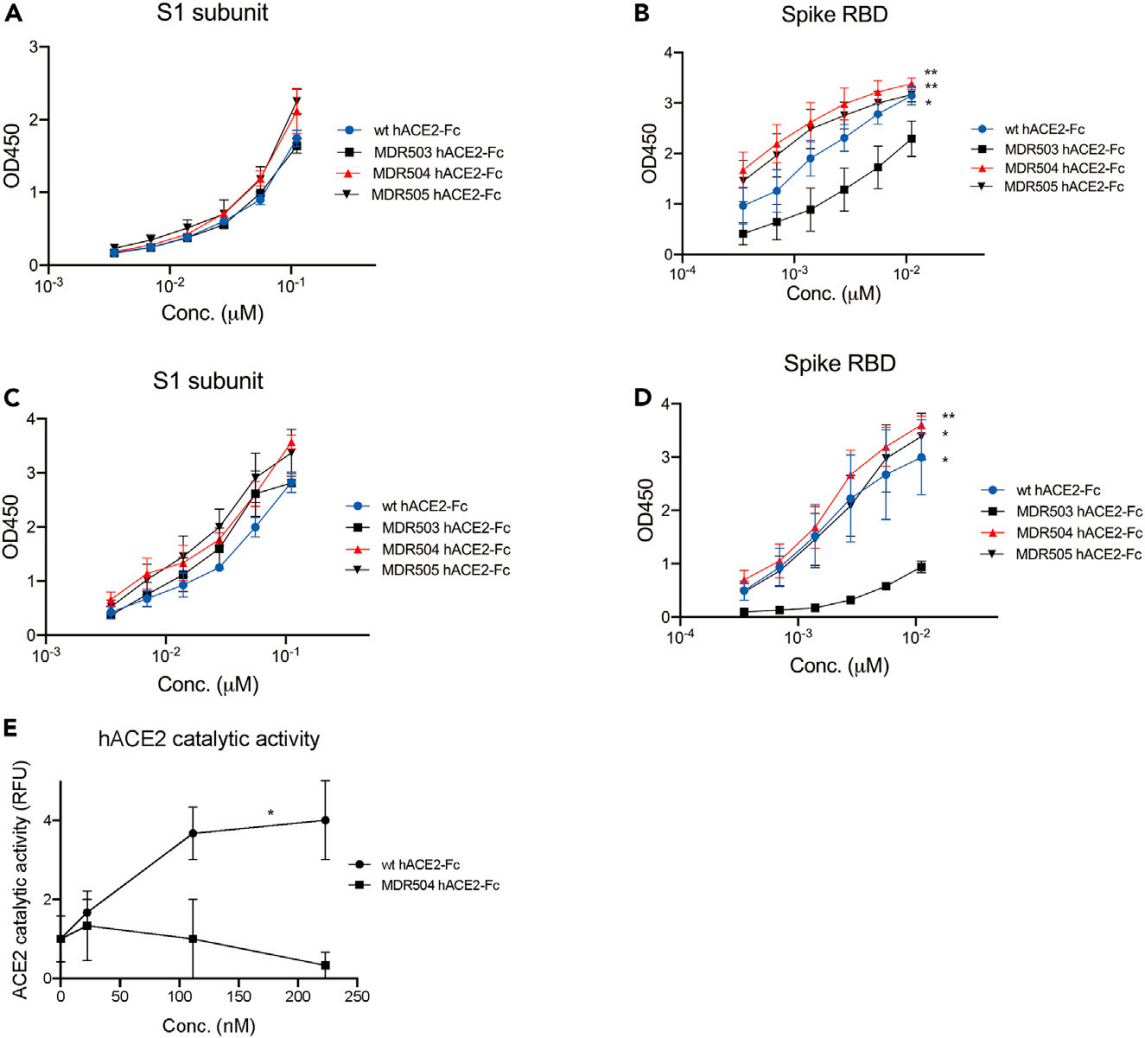
*In vitro* binding of MDR504 (A–D) Binding between S1 subunit from RayBiotech at room temperature (A) or at 37°C (C), or spike RBD from ATCC at room temperature (B) or at 37°C (D) and individual hACE2-Fc were assessed by ELISA (*n* = 3, representative from 2 independent experiments). (E) Catalytic activity of WT and the MDR504 mutant in a fluorogenic peptide assay (*n* = 3, representative from 2 independent experiments). Data are represented as mean ± SEM. Significant differences are designated using one-way ANOVA followed by Tukey's multiple comparisons test (B, D) and unpaired t test (E) based on the area under the curve (AUC). (B, D) ∗p<0.05; ∗∗p<0.01 (compared to MDR503), (E) ∗p<0.05 (compared to MDR504).

In experiments performed at room temperature, we observed higher binding of the MDR504 and MDR505 ACE2-Fc compared to WT ACE2-Fc (Figures 1A and 1B). This increase in binding was more dramatic when assayed at 37°C (Figures 1C and 1D) where binding of the MDR504 mutant was superior against the monomeric spike receptor-binding domain (RBD) protein from ATCC. To test whether the MDR504 mutant lacked ACE2 catalytic activity, we performed an *in vitro* catalysis assay using a fluorogenic peptide that contains an ACE2 cleavage site. Using this assay, WT ACE2 showed substantial catalysis of the peptide whereas the MDR504 mutant completely lacked any detectable activity (Figure 1E).

**Figure 2:**
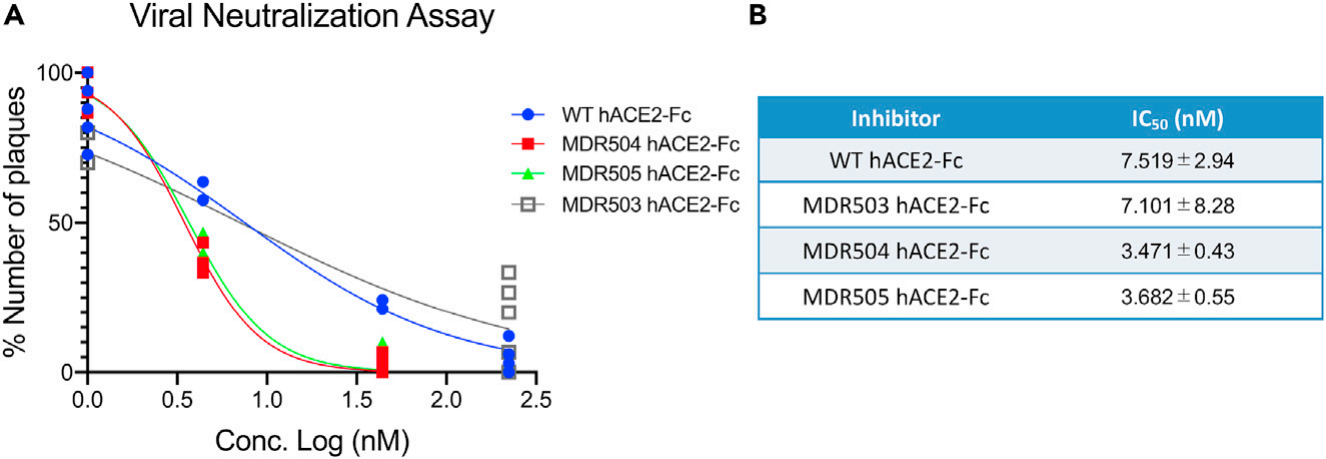
*In vitro* viral neutralization by plaque assay (A) SARS-CoV-2 neutralization with WT hACE2-Fc, MDR503, MDR504, and MDR505 mutant were analyzed by plaque assay (*n* = 3, cumulative data of two separate experiments). (B) Calculated IC_50_ (mean ± s.d.) of each construct in the plaque assay.

Using a pseudotyped model of SARS-CoV-2, we found superior neutralization with the MDR504 and MDR505 compared to WT hACE2 Fc (Figures S2A and S2B). The dose-response curve showed excellent neutralization by MDR505 (WT hACE2; 8.58 nM vs MDR505 hACE2; 2.01 nM) (Figures S2C and S2D). Next, we examined neutralization of SARS-CoV-2 (Wuhan-Hu-1; GenBank: MN_908947) using a plaque assay in Vero E6 cells. 50 μg/mL WT hACE2-Fc completely neutralized SARS-CoV-2 *in vitro* (data not shown). Thus, we compared the wild-type protein to the MDR503, MDR504, and MDR505 at lower concentrations. Here, MDR504 and MDR505 efficiently neutralized virus infection and had lower IC50s than the wild-type protein (Figures 2A and 2B).

We deposited MDR504 in the COVID database: https://covic.lji.org/databases/ and they found that this protein has a Kd of 1.62 × 10^−9^ using the full-length D614 ectodomain and a Kd of 8.03 × 10^−10^ against the full-length D614G ectodomain (Table S1). Thus, the MDR504 and MDR505 mutation may have a structural basis for enhanced neutralization over WT hACE2-Fc and that will be the subject of future research. Based on the lower IC50 of MDR504 against authentic SARS-CoV-2 virus (Wuhan-Hu-1; GenBank: MN_908947), we prioritized this protein for *in vivo* testing as well as *in vitro* binding to SARS-CoV-2 variants (Table S2).

### *In vivo* studies of MDR504

**Figure 3:**
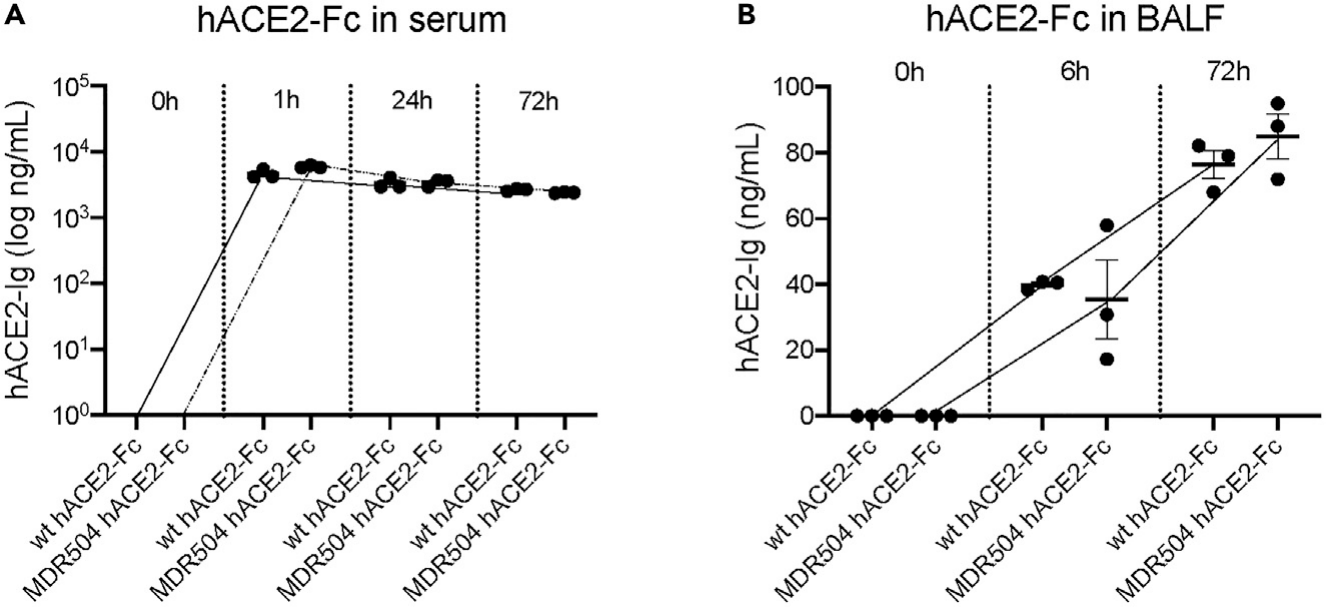
*In vivo* PK of MDR504 *In vivo* pharmacokinetics in 6- to 10-week-old male wild-type C57BL/6J mice of the WT and MDR504 mutant hACE2-Fc were assayed after intravenous injection of 4 mg/kg body weight of the protein in serum (A) and BAL fluid (B) (*n* = 3, single experiment). Data are represented as mean ± SEM.

**Figure 4:**
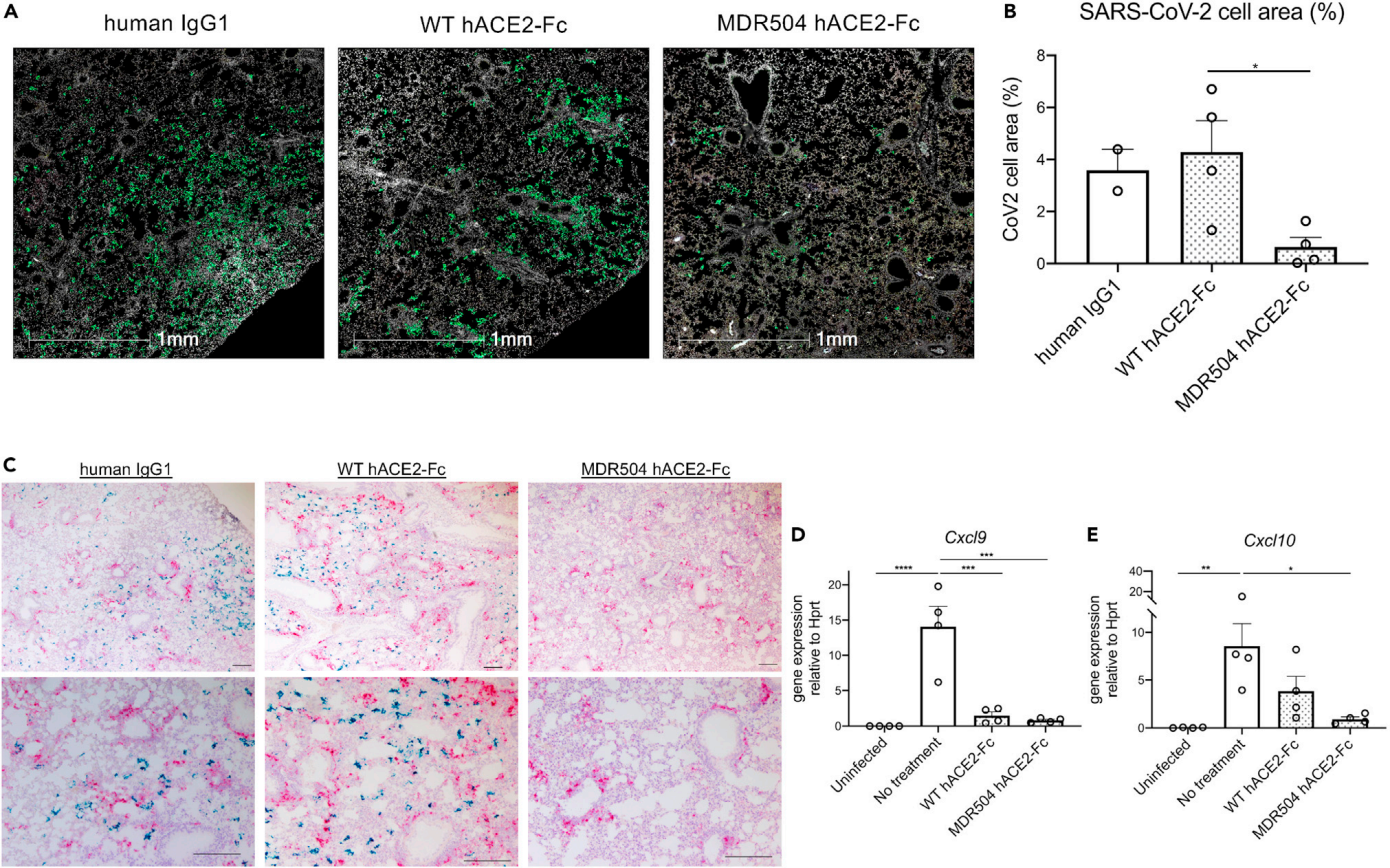
*In vivo* prophylaxis with MDR504 Four days post oropharyngeal inoculation of Ad5-hACE2, and 4 h before SARS-CoV-2 challenge, 6- to 10-week-old male wild-type C57BL/6J mice were treated with 15 mg/kg body weight human polyclonal IgG1, WT hACE2-Fc, or MDR504 hACE2-Fc intravenously. Three days later, mice were euthanized in ABSL3. SARS-CoV-2 infection was assayed by immunohistochemistry (White: DAPI, Green: SARS-CoV-2) (A, B) (scale bar, 1 mm). RNAscope was also performed on the same tissue (Red: *Cxcl9*, Blue: *SARS-CoV-2*) (C) (scale bar, 500 μm), and *Cxcl9* and *Cxcl10* gene expression were assessed by real-time RT-PCR (D, E). Data are represented as mean ± SEM. Significant differences are designated using one-way ANOVA followed by Tukey's multiple comparisons test. ∗p<0.05, ∗∗p<0.01, ∗∗∗p<0.001, ∗∗∗∗p<0.0001 (*n* = 3–4, representative from 2 independent experiments).

After a single IV injection, the MDR504 mutant had similar serum stability as WT hACE2-Fc (Figure 3A), and we detected higher tendency in the epithelial lining fluid of the lung after parenteral administration to C57BL/6J mice, but that was not significant (Figure 3B). The MDR504 mutant had a slightly higher peak concentration in serum and a half-life of approximately 145 h. We next tested the efficacy of engineered hACE2-Fc in our recently established mild to moderate COVID-19 mouse model, namely, SARS-CoV-2-infected mice that are induced to express hACE2 via delivery of adeno 5-derived hACE2 viruses to their lung (Ad5-hACE2) (Han et al., 2020; Sun et al., 2020). Mice were untreated or dosed intravenously with 15 mg/kg with human polyclonal IgG1, WT hACE2-Fc, or MDR504 four hours prior to SARS-CoV-2 infection for a prophylaxis model. Histological analysis of untreated, human IgG1-treated, and WT hACE2-Fc-treated Ad5-hACE2 mice showed similar widespread SARS-CoV-2 infection in the distal lung with approximately 4% of the cell area infected (naive lung (%); 4.32 ± 0.37, human IgG1-treated lung (%); 3.59 ± 0.80, WT hACE2-Fc treated lung (%); 4.29 ± 0.80). In contrast, MDR504 had significantly less infected cell area positive for SARS-CoV-2 staining (MDR504 hACE2-Fc-treated lung (%); 0.64 ± 0.37) (Figures 4A and 4B). In addition, we performed RNA scope, which is an RNA ISH technology with a unique probe design strategy that allows simultaneous signal amplification and background suppression to achieve single-molecule visualization while preserving tissue morphology. RNA scope for viral RNA showed significant fewer infected cells in MDR504 treatment (Figure 4C). We checked the viral load but it was not significant due to the sample variances (Figure S3). We speculate some of this signal likely represented the viral inoculum. Additionally, MDR504 lacks FCR binding and thus viral RNA may be sequestered virus whereas the viral protein staining is a more accurate measure of the number of lung cells actually infected. Of note, infection with SARS-CoV-2 induces interferon-stimulated chemokines such as *Cxcl9* and *Cxcl10* similar to infected human lung (Liao et al., 2020). Notably, in MDR504-treated Ad5-hACE2 mice, the *Cxcl9* gene expression was relatively reduced by RNAscope (Figure 4C) and the *Cxcl9* and *Cxcl10* gene expression was substantially downregulated by RT-PCR (Figures 4D and 4E).

### Effects of MDR504 in a treatment model of SARS-CoV-2 pulmonary infection

**Figure 5:**
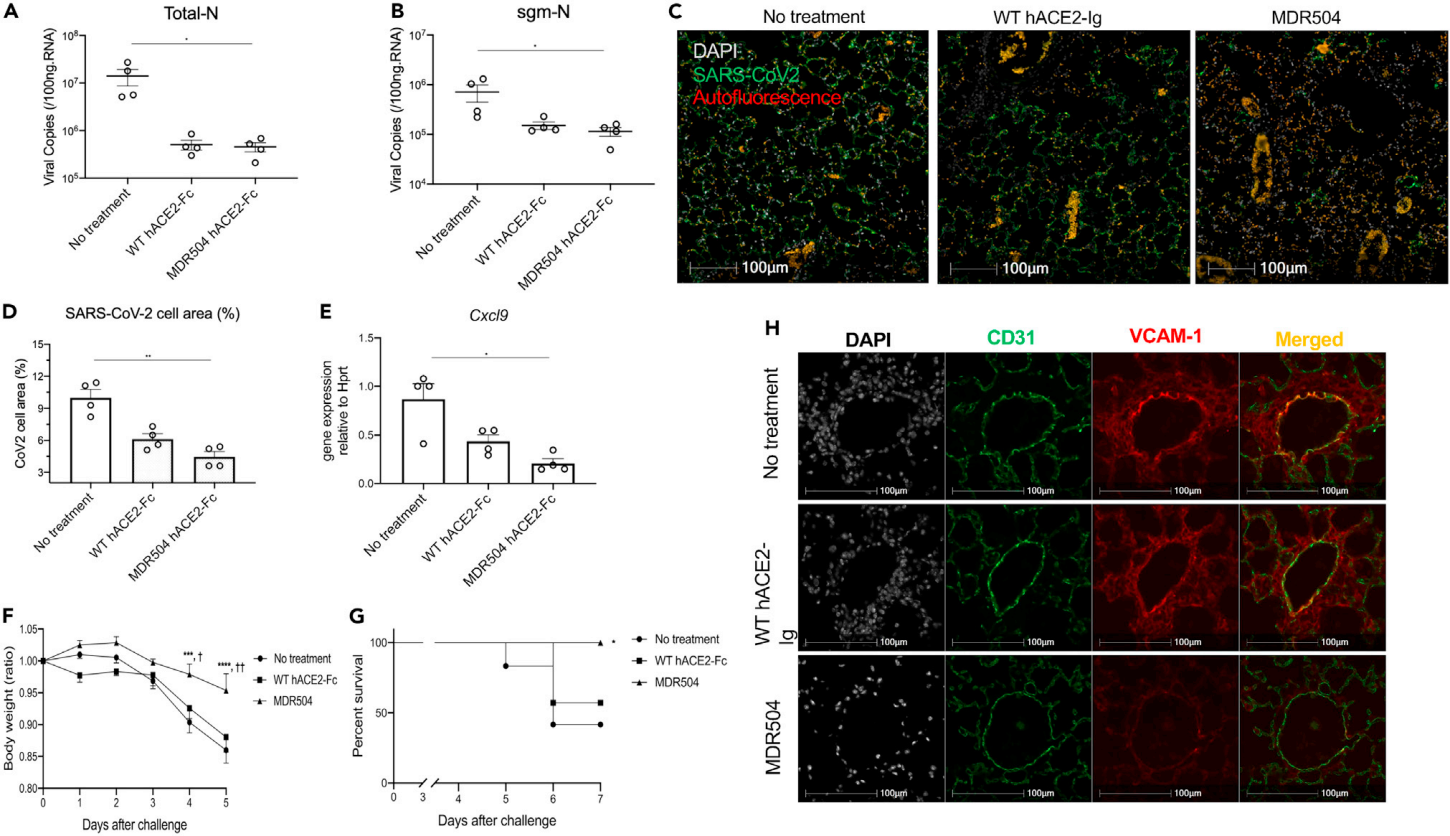
*In vivo* efficacy of MDR504 in the K18-ACE2 COVID-19 model 6- to 10-week-old male K18-ACE2 mice were treated with 30 mg/kg body weight WT hACE2-Fc, or MDR504 hACE2-Fc intravenously 4 h post SARS-CoV-2 challenge. Three days later, mice were euthanized in ABSL3. (A–D) Total-N viral copies, (B) subgenomic-N viral copies, and (C) SARS-CoV-2 staining and (D) quantitative analysis of the staining area (%) in lungs assayed by immunohistochemistry three days post challenge are shown. (E) *Cxcl9* gene expression as measured by real-time RT-PCR. Significant differences are designated using Kruskal-Wallis test followed by Dunn's multiple comparisons test. ∗p<0.05, ∗∗p<0.01. (F) Weight loss in the following mice: MDR504 (n = 8), WT hACE2-Fc (n = 7). or vehicle (n = 12), ^†^p <0.05 and ^††^p <0.01 (vs WT hACE2-Fc), ∗∗∗p <0.001 and ∗∗∗∗p <0.0001 (vs No treatment) by two-way ANOVA (cumulative data of two separate experiments). (G) Survival in the flowing groups: MDR504 (n = 8), WT hACE2-Fc (n = 7), or vehicle (n = 12). p<0.05 log rank test (cumulative data of two separate experiments). (H) Immunofluorescent staining for CD31 and Vcam1 including of DAPI was performed to detect endothelial dysfunction. Data are represented as mean ± SEM (A, B, D, E, F).([C] and [H]: scale bar, 100 μm).

We next examined MDR504 in treatment models in which we administered the proteins post SARS-CoV-2 challenge. Based on more potent binding of the CHO protein by ELISA (Figure S4A) and improved pharmacokinetics (Figures S4B and S4C) of this molecule, we used CHO protein in the treatment model. We confirmed that MDR504 protein produced in CHO had the nearly identical molecular weight as the protein produced in 293T cells (Figure S4D). To evaluate treatment, we tested MDR504 in a severe COVID-19 mouse model, namely, K18-hACE2 transgenic mice of SARS-CoV-2 infection model (Yinda et al., 2021). Before starting the experiment, we confirmed there is significant viral replication in the lung as measured by ∼ 10^4^ subgenomic RNA copies in the lung four hours after intranasal administration (Figure S5). As K18-hACE2 mice support significantly higher viral replication than the Ad5-hACE2 model, we administered MDR504 or control 4 h after viral infection and increased the dose to 30 mg/kg body weight. In the high-dose SARS-CoV-2 model, MDR504 substantially suppressed viral load as measured by total and subgenomic N gene expression (Figures 5A and 5B), dramatically reduced number of the infection cells in the lung, evidenced by SARS-CoV-2 viral staining, a method directly evaluating the severity of the lung infection (Figures 5C and 5D) (Han et al., 2020), and inhibited *Cxcl9* gene expression (Figure 5E), although this was not significant compared to WT hACE2-Fc. *Cxcl10* was also assessed but it was not different among the samples (Figure S6). This could be due to the rapid induction of *Cxcl10* by 4 h in the treatment model. Notably, MDR504 significantly improved and ameliorated weight loss (Figure 5F) and survival (Figure 5G) in the K18-ACE2 model. Pulmonary endothelial injury is a hallmark of pathological change in patients with severe COVID-19 (Ackermann et al., 2020; O'Sullivan et al., 2020). Vcam1 is a surrogate marker for assessing endothelial dysfunction (Liao, 2013). MDR504 treatment significantly downregulated the expression of the Vcam1, on pulmonary endothelial cells (Figure 5H), indicating the treatment protects the pulmonary endothelial cells from SARS-CoV-2 infection-mediated dysfunction and injury, which could be one of the multiple reasons for the benefit on the prolonged survival.

### Activity of MDR504 against several variants of concern

**Figure 6:**
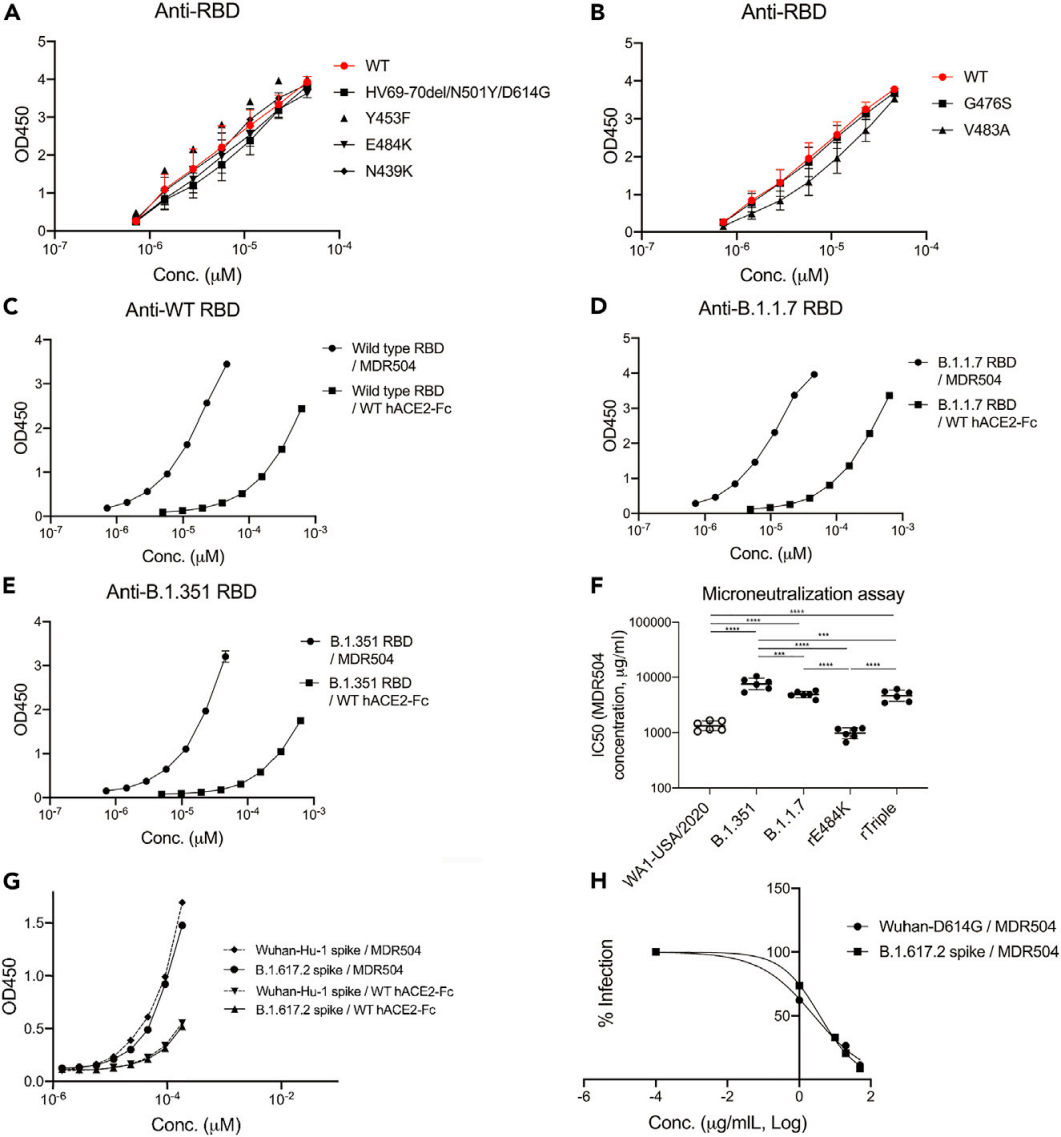
*In vitro* binding of MDR504 to SARS-CoV-2 variant proteins (A and B) Dose-dependent binding of MDR504 to several spike variants. (C, D, and E) MDR504 versus WT hACE2-Fc binding to RBD proteins encoding the WT strain (C), (B)1.1.7 (D) and (B)1.351 variants (E) (*n* = 3, representative from 2 independent experiments). (F) Microneutralization assays were performed three times on Vero E6 cells from different passages with two different batches of MDR504 for all viruses. rTriple is defined as the SARS-CoV-2 variant with the mutations of K417N, E484K, and N501Y. Data are presented as geometric mean ± geometric SD (*n* = 6). (G) Compared to the WT hACE2-Fc, MDR504 showed a better binding with WT spike and (B)1.617.2 variant. (H) Pseudovirus neutralization assay also depicts comparable neutralization of MDR504 with WT spike or (B)1.617.2 variant (*n* = 3). Data are represented as mean ± SEM. Significant differences are designated using one-way ANOVA followed by Tukey's multiple comparisons test. ∗∗∗p<0.001, ∗∗∗∗p<0.0001.

Lastly, we tested MDR504 binding to several spike variants (Table S2) that have been associated with reduced binding by casirivimab, imdevimab, or bamlanivimab (Li et al., 2020; Liu et al., 2020; Starr et al., 2021). MDR504 retained pM binding to these variants (Figure 6A) with only mildly reduced binding to V483A (Figure 6B). We observed enhanced binding of MDR504 to ACE2 to variants Y453F as well as HV69-70del/N501Y/D614G that were identified in the SARS-CoV-2 variant (Figure 6A). We also observed increased binding to the alpha variant, first detected in the UK (B.1.1.7) (Figure 6D), that was significantly shifted to the left compared to wild-type ACE2 (Figure 6C). Binding to the beta variant, first detected in South Africa (B.1.351) (Figure 6E), was also significantly higher with MDR504 versus wild-type ACE2. Moreover, using a microneutralization assay, we established the IC50 of MDR504 against different SARS-CoV-2 variants including two variants of concern as well as recombinant viruses that have either the E484K (rE484K) or K417N, E484K and N501Y (rTriple) (Figure 6F) mutations. Compared to the WT hACE2-Fc, MDR504 showed enhanced binding with WT spike and the B.1.617.2 variant (Figure 6G). A pseudovirus neutralization assay also showed comparable neutralization of MDR504 between WT spike and the delta variant (B.1.617.2) (Figure 6H).

## Discussion

ACE2 is expressed in the nasal respiratory epithelium, in the conducting airway in type II pneumocytes (Hou et al., 2020), and elsewhere (Hoffmann et al., 2020). One potential mechanism that has made SARS-CoV-2 more infectious than the 2002 SARS-CoV epidemic is the higher affinity of SARS-CoV-2 spike protein to the human ACE2 (Shang et al., 2020; Yan et al., 2020). However, this increased affinity represents a potential therapeutic target to block viral entry. Recent surface plasmon resonance assays with soluble ACE2 binding SARS-CoV-2 spike protein had a binding affinity in the nM to pM range (Lei et al., 2020). Thus, a soluble version of ACE2 may have potent viral neutralization activity. ACE2 is a type I transmembrane protein with a 740 amino acid ectodomain (Crackower et al., 2002). Soluble ACE2 has been shown to bind to SARS-CoV and SARS-CoV-2 spike proteins and block viral entry (Monteil et al., 2020). Soluble ACE2 has been administered to humans with pulmonary hypertension (Hemnes et al., 2018) and acute respiratory distress syndrome (ARDS) (Khan et al., 2017) at dose ranges of 0.1–0.8 mg/kg and shown to be well tolerated. However, the pharmacokinetic/pharmacodynamic (PK/PD) of soluble ACE2 is not ideal for sustained viral neutralization *in vivo* and soluble ACE2 is not engineered to be transported from the circulation into the epithelial lining fluid of the lung. ACE2-IgG Fc fusion proteins have been reported to also bind virus and neutralize SARS-CoV-2 pseudoviruses *in vitro* (Lei et al., 2020). Moreover, several mutants in the catalytic domain have been reported that also bind and neutralize virus (Lei et al., 2020). However, ACE2-IgG Fc fusion proteins retain FcRγ binding which may compromise serum stability or activate FcRγ on myeloid cells, which may be problematic in COVID-19.

To this end, we designed and produced hACE2-Fc fusions with mutations in the hACE2 catalytic domain as well as in the IgG1 constant region to abrogate FcRγ binding with a LALA mutation, but retain binding to the neonatal Fc receptor which is important for serum stability (Arduin et al., 2015) as well as transport into the lung (Kolls et al., 1995). All constructs were efficiently secreted after transient transfection in 293T or CHO cells and purified using protein G resin. All proteins bound monomeric SARS-CoV-2 receptor binding domain as well as trimeric spike protein (Amanat et al., 2020). Initial studies were done at 50 μg/mL (223 nM) based on studies with Pavalizumab, an anti-RSV monoclonal antibody, showed that effective anti-RSV trough concentrations *in vivo* were ∼40 μg/mL (Beeler and van Wyke Coelingh, 1989). Using both pseudovirus and authentic virus, MDR504 showed efficacy against SARS-CoV-2 infection *in vitro* and *in vivo*. Moreover, MDR504 appears to maintain potency to several variants of concern.

At this time, the structural basis for this remains unclear. Cryo-EM studies of SARS-CoV-2 RBD have been shown to bind the NH2 terminus of human ACE2 (Lan et al., 2020). However, the RBD also binds to residues K353, G354, and D355 (Lan et al., 2020) and thus it is possible that the MDR504 mutation in the catalytic domain affects this binding. The other reported hACE2-Fc mutants showed equivalent binding and pseudovirus neutralization (Lei et al., 2020). The authors of this paper made H374N and H378N mutations that have putatively reduced ACE2 catalytic activity but that was not specifically assayed as part of their study (Lei et al., 2020). However, our IC_50_ data are in agreement with their calculated *K*_*d*_ using surface plasmon resonance where the *K*_*d*_ was reported to be 11.2 nM (Lei et al., 2020). Prior structural studies suggest that H345 is not only in the catalytic pocket but also very close to the RBD binding site. Analysis in Raptor suggests that H345A may reduce constraint of the alpha-helix that interacts with SARS-CoV-2 spike RBD. Note that the H345A mutation has reduced catalytic activity in the fluorogenic peptide assay used here but retains activity against full-length substrate.

Taken together, this reagent may be useful as pre- and post-exposure prophylaxis or as therapy for COVID-19 as well as emerging variants of SARS-CoV-2. This technology may also complement vaccine technology, as it may be useful in subjects that may not be good candidates for vaccines such as patients with hematologic or other malignancies, or those that are undergoing immunosuppressive therapy for organ transplantation or autoimmune disease.

### Limitations of the study

This study has several limitations. First, we did not formally evaluate if the mutant ACE2 constructs are immunogenic after single IV administration. Secondly, the mode of viral inoculation in K18-ACE2 mice results in rapid infection of the lower respiratory tract which likely reduces the duration of the therapeutic window in this animal model.

## STAR★Methods

### Key resources table

**Table:** 

REAGENT or RESOURCE	SOURCE	IDENTIFIER
**Antibodies**		

InVivoMAb human IgG1 isotype control	Bio X Cell	BE0297; AB_2687817
Goat Anti-Human IgG Fc, Multi-Species SP ads-HRP	Southern Biotech	2014-05; AB_2795580
Human IgG Fc	Biolegend	410701; AB_2565624
goat anti-human IgG HRP	Southern Biotech	2040-05; AB_2795644
Polyclonal Anti-SARS Coronavirus	BEI	NR-10361
Anti-CD31	R&D	AF-3628
Anti-VCAM-1	Abcam	Ab134047
Goat anti-Guinea Pig	Invitrogen	A-11073; AB_2534117
Donkey anti-Goat	Invitrogen	A-11055; AB_2534102
Donkey anti-Rabbit	Invitrogen	A-31573; AB_2536183

**Bacterial and virus strains**		

SARS-Related Coronavirus 2, Isolate USA-WA1/2020	BEI RESOURCES	NR-52281
human ACE2 Adenovirus	Vector Biolabs	ADV-200183

**Chemicals, peptides, and recombinant proteins**		

10x Tris/Glycine/SDS	BioRad	1610732
Precision Plus Protein™ Dual Color Standards, 500 μl	BioRad	1610374
10% Mini-PROTEAN® TGX Stain-Free™ Precast Gels	BioRad	456-8033
Cell Lysis Buffer (10x)	Cell Signaling Technology	#9803
Thermo Scientific SuperSignal West Pico Chemiluminescent Substrate	Thermo Fisher Scientific	37048
Recombinant SARS-CoV-2 Spike Protein, S1 Subunit, Host Cell Receptor Binding Domain (RBD)	Raybiotech	230-01102
Spike Glycoprotein Receptor Binding Domain (RBD) from SARS-Related Coronavirus 2, Wuhan-Hu-1, Recombinant from HEK293 Cells	BEI RESOURCES	NR-52306
Lipofectamine™ 3000 Transfection Reagent	Invitrogen	L3000015
TRIzol™ Reagent	ThermoFisher Scientific	15596018
taqman gene expresion master mix	ThermoFisher Scientific	4369510
iscript Reverse Transcription Supermix for RT qPCR	Bio-Rad	1708841
Recombinant SARS-CoV-2 G476S Spike RBD His-tag Protein, CF	R&D	10627-CV-100
Recombinant SARS-CoV-2 V483A Spike RBD His-tag Protein, CF	R&D	10628-CV-100
Recombinant SARS-CoV-2 Spike RBD His-tag Protein, CF	R&D	10500-CV-100
SARS-CoV-2 (COVID-19) S protein RBD (E484K), His Tag (MALS verified)	Acro Biosystem	SRD-C52H3-100
SARS-CoV-2 (COVID-19) S protein RBD (N439K), His Tag (MALS verified)	Acro Biosystem	SRD-C52Hg-100
SARS-CoV-2 (COVID-19) S protein RBD (Y453F), His Tag (MALS verified)	Acro Biosystem	SRD-C52Hk-100
SARS-CoV-2 (COVID-19) S protein (HV69-70del, N501Y, D614G), His Tag	Acro Biosystem	S1N-C52Hk-100
SARS-CoV-2 (COVID-19) S protein RBD, His Tag	Acro Biosystem	SPD-C52H1-200
Spike Glycoprotein (Stabilized) from SARS-Related Coronavirus 2, Wuhan-Hu-1 with C-Terminal Histidine Tag	BEI RESOURCES	NR-52397
Spike Glycoprotein (Stabilized) from SARS-Related Coronavirus 2, Delta Variant with C-Terminal Histidine and Avi Tags	BEI RESOURCES	NR-55614

**Critical commercial assays**		

IgG (Total) Mouse Uncoated ELISA Kit	Thermo Fisher Scientific	88-50400-88
Pierce™ BCA Protein Assay Kit	Thermo Fisher Scientific	23225
RNeasy Plus Mini Kit	QIAGEN	74134
NAb Protein G Spin Columns	Thermo Fisher Scientific	89953
Amicon® Ultra-15 Centrifugal Filter Unit	Millipore Sigma	UFC901024
RNAscope® Probe V-*nCoV2019-S*	Advanced Cell Diagnostics	848561
RNAscope® Probe Mm-*Cxcl9*-C2	Advanced Cell Diagnostics	489341-C2

**Experimental models: Cell lines**		

293T cells	Invitrogen	N/A

**Experimental models: Organisms/strains**		

C57BL/6J mice	The Jackson Laboratory	000664
B6.Cg-Tg(K18-ACE2)2Prlmn/J mice	The Jackson Laboratory	034860

**Oligonucleotides**		

Cxcl9 Mm00434946_m1	ThermoFisher Scientific	# 4331182
Cxcl10 Mm00445235_m1	ThermoFisher Scientific	# 4331182
Primer: subgenomic-N QPCR: F; 5’-CGA TCT CTT GTA GAT CTG TTC TC-3′, R; 5′-GGT GAA CCA AGA CGC AGT AT-3′, P; 5′-56-FAM/TAA CCA GAA/ZEN/TGG AGA ACG CAG TGG G/36-TAMSp/-3′	This paper	N/A
Primer: Total-N QPCR: F; 5′-GTT TGG TGG ACC CTC AGA TT′, R; 5′-GGT GAA CCA AGA CGC AGT AT -3′, P; 5′-56-FAM/TA ACC AGA ATG GAG AAC GCA GTG GG/36-TAMSp-3′	This paper	N/A

**Recombinant DNA**		

iggk hACE2 ED-Ig_pcDNA3.1/Hygro(+)	GenScript	U414RFD030 _4
iggk hACE2 ED R273A Ig_pcDNA3.1/Hygro(+)	GenScript	U414RFD030 _2
iggk hACE2 ED H345A Ig_pcDNA3.1/Hygro(+)	GenScript	U414RFD030 _8
iggk hACE2 ED R273A H345A Ig_pcDNA3.1/Hygro(+)	GenScript	U414RFD030 _6

**Software and algorithms**		

Prism 9.0	GraphPad	N/A
Raptor X	Xu. et al, (2008)	http://raptorx.uchicago.edu/

### Resource availability

#### Lead contact

Further information and requests for resources and reagents should be directed to and will be fulfilled by the lead contact, Jay Kolls (jkolls1@tulane.edu).

#### Materials availability

Reagents such as DNA constructs generated in this study will be made available on request, but we may require a payment and/or a completed Materials Transfer Agreement from Tulane University.

### Experimental model and subject details

#### Mice

Male wild-type C57BL/6J mice or male K18-hACE2 transgenic mice 6-10-week-old were used for *in vivo* studies. The mice were bred in-house or purchased from The Jackson Laboratory. All experiments were performed using sex- and age-matched controls and approved by the Institutional Animal Care and Use Committee of Tulane University.

#### SARS-CoV-2 infection

SARS-CoV-2 is an isolate USA-WA1/2020, NR-52281 deposited by the Centers for Disease Control and Prevention and sold by BEI Resources, NIAID, NIH. We passaged the virus in VeroE6 cells in DMEM media with 2% FBS and sequenced the virus for verification as described previously (Han et al., 2020). Mice were intranasally infected by 2 X 10^5^ TCID50 SARS-CoV-2 in ABSL3.

#### *In vivo* prophylaxis and treatment model of MDR504

All animals were cared for in accordance with the NIH guide to Laboratory Animal Care. The Institutional Biosafety Committee approved the procedures of sample handling, inactivation, and removal from a BSL3 containment. For a prophylaxis model, we utilized the murine model of SARS-CoV-2 that C57BL/6J mice were first oropharyngeally inoculated with 1.5 × 10^9^ pfu adenovirus encoding human ACE2 (Vector Biosystems INC, Malvern, PA) as described our recent publication (Han et al., 2020). Four days later, mice received 2 × 10^5^ pfu of SARS-CoV-2 intranasally and were euthanized 72 h post infection by the ABSL3 staff. To evaluate MDR504, mice were dosed intravenously via retro-orbital with 15 mg/kg body weight polyclonal human IgG1, WT hACE2-Fc or MDR504 4 h prior to SARS-CoV-2 infection for prophylaxis model. As a treatment model of high viral load, we confirmed the efficacy using K18-hACE2 transgenic mice, in which we infected 2 × 10^5^ pfu SARS-CoV-2 and treated with the 30 mg/kg body weight of the antibodies 4 h post infection.

### Method details

#### Generation of different constructs of human ACE2 fusion proteins

The DNA sequences of the extracellular domains of ACE2 and IgG1Fc were synthesized by Genscript and cloned into pcDNA3.1. Iggk hACE2 ED R273A Ig is labeled as MDR503, Iggk hACE2 ED H345A Ig is labeled as MDR504, and Iggk hACE2 ED R273A H345A Ig is labeled as MDR505. Transient transfection was performed using Lipofectamine™3000 Transfection Reagent (Invitrogen) in 293T cells. The collected supernatants were collected and purified by protein G-sepharose (Thermo Fisher). Additionally, we utilized MDR504 produced in CHO cells (produced by Lake Pharma, Dallas TX). The concentration and purity were confirmed by measuring the UV absorbance at wavelength of 280 nm, BCA assay (Thermo Fisher) and human IgG ELISA (Thermo Fisher).

#### Western blotting

Following the removal of the supernatants, cell lysates were dissolved in 50 mg/mL cell lysis buffer (Cell signaling) containing protease inhibitors (Thermo Scientific) and 1 mM PMSF. BCA assay was performed to quantify protein and 5.0 μg protein was used for Western blotting. Western blots were performed using 7.5% SDS-PAGE gels (Bio-Rad) under the non-reducing or reducing condition with 2.5% 2-mercaptoethanol and transferred to PVDF membranes. The blot was probed with goat anti-human IgG-HRP (Southern Biotech). After incubation with IgG-HRP-conjugated anti-human antibody, membranes were washed and incubated with SuperSignal West Pico Chemiluminescent Substrate (Thermo Scientific). Signal was detected using Bio-Rad ChemiDoc MP imaging system.

#### ELISA for human ACE2 and spike proteins

ELISA plates were coated with 2 μg/mL recombinant S1 subunit, receptor binding domain (RBD) from RayBiotech (Cat #230-01102, Accession number; QHD43416) or spike glycoprotein RBD from ATCC (BEI RESOURCES, Cat # NR-52306) overnight at 4°C. Coated plates were washed with washing buffer (0.05% Tween 20 in PBS), blocked for 2 h at room temperature with blocking buffer (1% BSA and 0.1% Tween 20 in PBS), and washed before the addition of the supernatants or cell lysates from transfected 293T cells. After 2 h incubation at RT, or 1 h incubation at 37°C, the plates were washed and incubated with goat anti-human IgG conjugated with horseradish peroxidase (Southern Biotech) diluted 1/5,000 in assay diluent (0.5% BSA and 0.05% Tween 20 in PBS) for 1 h at RT, or for 30 min at 37°C, TMB peroxidase substrate (Southern Biotech) was added to each well. Absorbance was read at 450 nm on a microplate reader (BioTek). Various mutant spike variants were examined for binding with WT hACE2-Fc and MDR504 (Table S2).

#### Pseudovirus production

The SARS-CoV-2 pseudovirus was generated using the following plasmids: The spike protein of SARS-CoV-2 (pcDNA3.1[+]-SARS2-S) and the HIV-1 pro-viral vector pNL4-3.Luc.R-E− which were obtained through the NIH AIDS Research and Reference Reagent Program. The pseudovirus were produced by transient co-transfection of 293T cells using a polyethyleneimine (PEI)-based transfection protocol. Five hours after transfection, cells were washed with phosphate-buffered saline (PBS), and 20 mL of fresh media was added to each 150 mm plate. Twenty-four hours post transfection, the supernatant was collected and filtered through a 0.45 μM pore size filter and stored at 4°C prior to use.

#### Pseudovirus neutralization assay

Targeted 293T cells were transfected with pcDNA3.1(+)-humanACE2 and pCSDest-TMPRSS2 plasmids for 6 h. The cells were then trypsinized and seeded 1x10^5^ cells/well in DMEM complete into 96-well plates (100 μL/well) then incubated for 16 h at 37°C and 5% CO_2_. SARS-CoV-2 pseudovirus was incubated with the test samples at room temperature for 1 h, and then added to the target cells in 96-well plates. Plates were incubated for 48 h at 37°C, 5% CO_2_ and levels of viral infection were determined by luminescence using the neolite reporter gene assay system (PerkinElmer). Virus alone was used as a control and data was normalized to the control. For B.1.617.2 neutralization, activity was measured in a single-round-of-infection assay with a SARS-CoV-2 pseudoviruses. SARS-CoV-2 pseudoviruses were produced by co-transfection of HEK293T cells with 4 plasmids: an expression plasmid bearing codon-optimized SARS-CoV-2 full-length S plasmid, a packaging plasmid pCMVDR8.2, a luciferase reporter plasmid pHR'CMV-Luc (40) and a TMPRSS2 plasmid (41). Plasmids were kind gifts from Barney Graham at VRC and David Montefiori at Duke U. Transfected 293T cells were cultured for 3 days. The supernatant fluids were collected, clarified, filtered and aliquoted. Virus stocks were frozen at−80°C. Pseudoviruses were mixed with serial dilutions of MDR504. We also incubated the same pseudoviruses with serial dilutions of our internal standard serum as a control. After 1 h incubation we added the mixtures to monolayers of ACE2-overexpressing CHO cells in triplicate. Three days post infection, cells were lysed, and luciferase activity was measured with the Bright GLo Luciferase Assay System (Promega). Relative light units (RLU) were measured in a Biotek Synergy H1 Luminometer. The RLU data were analyzed in GraphPad prism to determine IC50 for each reagent and plasma.

#### Human ACE2 neutralization of SARS-CoV-2 by plaque assay

Vero E6 cells were plated in a 6 well plate at 8 x 10^5^ cells per well and incubated overnight. Each construct of hACE2-Fc was preincubated with SARS-CoV-2 (L strain of Wuhan-Hu-1; GenBank: MN_908947) for 10 min before infection in 1 mL media. The cells were washed once with PBS and infected at a MOI of 0.01 with the compounds for 1 h. Following infection, the supernatants containing the virus and compounds were removed and 3 mL overlay media containing each compound were added to the wells and incubated for additional 4 days. Post infection, the cells were fixed and stained to visualize plaques.

#### Pharmacokinetics study

C57BL/6J mice were injected with 4 mg/kg body weight WT hACE2-Fc or the MDR504 mutant intravenously via retro-orbital and serum was collected at 0, 1, 24, or 72 h. Mice were euthanized at 6h and 72 h and underwent bronchoalveolar lavage (BAL) to measure hACE2-Fc in the epithelial lining fluid of the lung. The concentration of hACE2-Fc was analyzed by detecting human IgG-Fc by ELISA. We used purified anti-human IgG Fc antibody (Biolegend) as a capture antibody and anti-Human IgG Fc, Multi-Species SP ads-HRP (SouthernBiotech) as a detection antibody and the other procedure is same as mentioned above.

#### Viral quantification and endothelial injury analysis by immunohistochemistry

Lung tissues were collected in Zinc formalin (Anatech), embedded in paraffin and 5 μm thick sections were cut, adhered to charged glass slides, baked overnight at 56°C and passed through Xylene, graded ethanol, and double distilled water to remove paraffin and rehydrate tissue sections. A microwave was used for heat induced epitope retrieval. Slides were heated in a high pH solution (Vector Labs H-3301), rinsed in hot water and transferred to a heated low pH solution (Vector Labs H-3300) where they were allowed to cool to room temperature. Sections were washed in a solution of phosphate-bufferred saline and fish gelatin (PBS-FSG) and transferred to a humidified chamber. Tissue was blocked with 10% normal goat serum (NGS) for 40 min, followed by 60 min incubation with the first antibodies (Table S3). Slides were transferred to the humidified chamber and incubated, for 40 min, with secondary antibodies tagged with various Fluor fluorochromes and diluted to a working concentration of 2 μg/mL (Table S3). Slides were mounted using a homemade anti-quenching mounting media containing Mowiol (Calbiochem #475904) and DABCO (Sigma #D2522) and imaged with a Zeiss Axio Slide Scanner. Whole slide images of the lungs were analyzed with computer software (HALO, Indica Labs) using a tissue segmentation algorithm, trained with a deep convolution neural network (VGG), by a board-certified veterinary pathologist to recognize cellular inflammation within the lungs of mice.

#### Real-time RT-PCR

RNA was isolated with RNeasy Plus Mini Kit (QIAGEN) post phase separation using Trizol reagent (ThermoFisher), and cDNA was prepared using iScript reverse transcriptase master mix (Bio-Rad). Real-time RT-PCR was carried out with Bio-Rad CFX96 system using TaqMan PCR Master Mix (ThermoFisher Scientific) and *Cxcl9* or *Cxcl10* premixed primers/probe sets from Thermo Fisher Scientific. Subgenomic mRNA (sgmRNA) encoding the N gene or total N gene was quantified using a published assay (Wolfel et al., 2020). The viral copy numbers from the lung samples are represented as copies/100ng of RNA.

#### RNA scope

Z-fix-fixed and paraffin-embedded lung sections underwent *in situ* hybridization according to the manufacturer's instructions (Advanced Cell Diagnostics, Inc). Briefly, after hydrogen peroxide treatment, we performed target retrieval, created a hydrophobic barrier, and applied AP conjugated-*nCoV2019-S* probe (RNAscope® Probe V-*nCoV2019-S*, ACD cat# 848561) and *Cxcl9* probe (RNAscope® Probe Mm-*Cxcl9*-C2, ACD cat# 489341-C2) for hybridization. After hybridization, tissues were stained with 50% Hematoxylin.

#### 50% tissue culture infective dose (TCID_50_) calculation and *in vitro* microneutralization assay

To estimate the neutralizing efficiency of MDR504, *in vitro* microneutralization assays were performed. MDR504 was serially diluted 3-fold starting from 160 mg/mLl in Vero-E6-infection medium (DMEM+ 2% FBS+ 1% non-essential amino acids). The samples were incubated with 350 tissue culture infective dose 50 (TCID_50_) of virus for 1 h in an incubator at 37°C, 5% CO_2_ followed by incubation on pre-seeded Vero-E6 at 37°C for 48 h. Plates were fixed in 4% formaldehyde at 4°C overnight. For TCID_50_ calculation, the virus stock was serially diluted 10-fold starting with 1:10 dilution and incubated on Vero-E6 cells for 48 h followed by fixation in 4% Formaldehyde. The cells were washed with 1xPBS and permeabilized with 0.1% Triton X-100 in 1XPBS. The cells were washed again and blocked in 5% non-fat milk in 1Xpbs + 0.1% Tween-20 for 1 h at room temperature. After blocking, the cells were incubated with anti-SARS-CoV-2 NP and anti-spike monoclonal antibodies, mixed in 1:1 ratio, for 1.5 h at room temperature. Cells were washed in 1xPBS and incubated with 1:5000 diluted HRP-conjugated anti-mouse IgG secondary antibody for 1 h at RT followed by a brief PBS wash. Finally, 100μL tetramethyl benzidine (TMB) substrate was added and incubated at RT until blue color appeared, and the reaction was terminated with 50μL 1M H_2_SO_4_. Absorbance was recorded at 450nm and 650nm as a reference and percentage reduction in infection was calculated as compared to negative control.

#### SARS-CoV-2 viruses for microneutralization

SARS-CoV-2 isolates USA-WA1/2020 and B.1.1.7 were obtained from BEI resources. B.1.351 (hCoV-19/USA/MD-HP01542/2021 JHU) virus was a gift from Dr. Andrew Pekosz (Johns Hopkins Bloomberg School of Public Health). rE484K is a recombinant SARS-CoV-2 virus that is identical to USA-WA1/2020 except for the E484K mutation introduced in the spike RBD (provided by the Martinez laboratory). All SARS-CoV-2 viruses were expanded on Vero-E6 cells and sequenced using ARTIC protocol developed (https://artic.network/ncov-2019), as previously described (Ramanathan et al., 2021). Viruses were titrated by tissue culture infective dose 50 on Vero E6 cells for use with *in vitro* infections.

### Quantification and statistical analysis

Statistical analysis was performed with GraphPad Prism 8.0. p values <0.05 was evaluated statistically significant. Comparisons between two normally distributed groups were performed by simple 2-tailed unpaired student's t-test. For multiple groups comparisons, we used one-way or two-way ANOVA with Tukey's post-hoc analysis for normal distribution and if not, we used Kruskal-Wallis with Dunn's multiple comparison test. Values are represented as means ± SEM. p values are annotated as follows (∗) ≤0.05, (∗∗) ≤0.01, (∗∗∗) ≤0.001, and (∗∗∗∗) ≤0.0001.

## Data Availability

The datasets generated during this study are available at Zenodo with the following accession link: https://doi.org/10.5281/zenodo.5815410. Any further information and requests should be directed to and will be fulfilled by the Lead Contact, Jay Kolls (jkolls1@tulane.edu).
