# Supplementary material for: ACE2-IgG1 fusions with improved in vitro and in vivo activity against SARS-CoV-2
Source: iScience. 2021 Dec 20;25(1):103670. doi: 10.1016/j.isci.2021.103670 (PMC8686446; doi:10.1016/j.isci.2021.103670)
Supplement: Document S1. Figures S1–S6 and Table S1–S3 [file mmc1.pdf]

iScience, Volume *25*

Supplemental information

ACE2-IgG1 fusions with improved *in vitro* and *in vivo* activity against SARS-CoV-2

Naoki Iwanaga, Laura Cooper, Lijun Rong, Nicholas J. Maness, Brandon Beddingfield, Zhongnan Qin, Jackelyn Crabtree, Ralph A. Tripp, Haoran Yang, Robert Blair, Sonia Jangra, Adolfo García-Sastre, Michael Schotsaert, Sruti Chandra, James E. Robinson, Akhilesh Srivastava, Felix Rabito, Xuebin Qin, and Jay K. Kolls

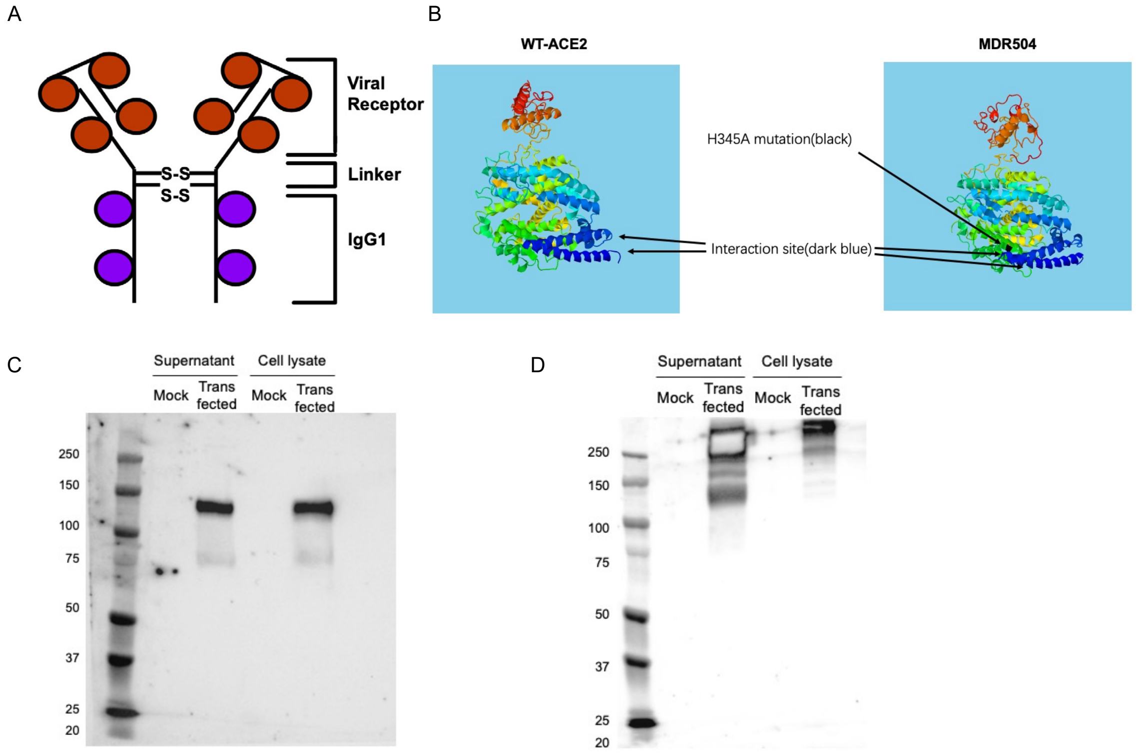

Figure S1. Construct of human ACE2 fusion protein. Related to Figure 1. (A) Schema of our engineered constructs of human ACE2 fused with IgG1Fc. (B) A schematic overview of the WT hACE2-Fc and MDR504 by Raptor X are shown. (C) Western blotting under reducing condition with 2.5% 2-mercaptoethanol of hACE2 expression in cell supernatants of transfected cells as well as cell lysates. (D) Western blotting of ACE2-IgG1 without reducing conditions.

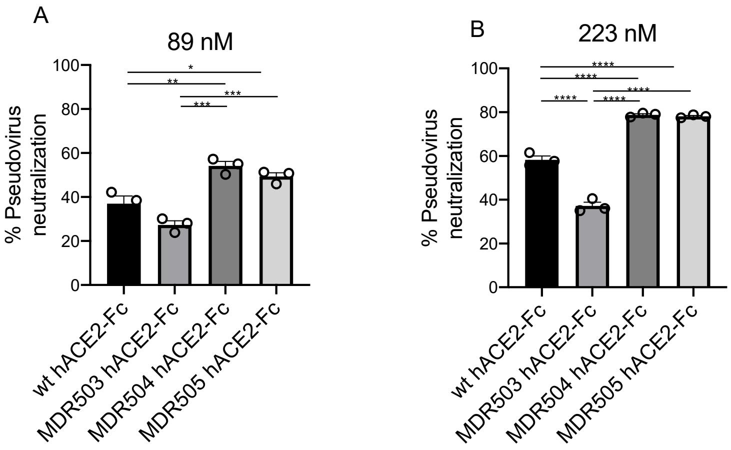

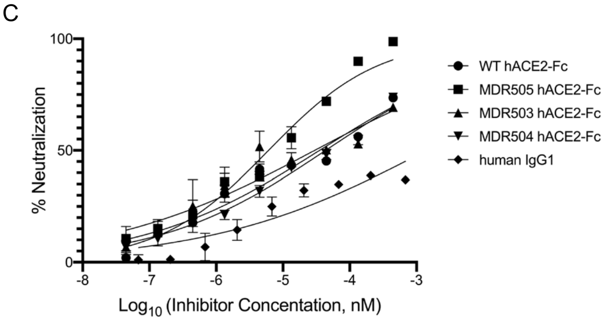

D

Inhibitor	IC_{50} (nM)
WT hACE2-Fc	8.58 ± 1.13
MDR505 hACE2-Fc	2.01 ± 1.23
MDR503 hACE2-Fc	7.34 ± 1.96
MDR504 hACE2-Fc	10.42 ± 4.07
human IgG1	21.66 ± 11.14

Figure S2. *In vitro* neutralization using pseudovirus. Related to Figure 2. Neutralization of pseudovirus were compared among the different constructs in (A) 89 nM and (B) 223 nM of hACE2-IgG1 concentration. Data are represented as mean +/- SEM. Significant differences are designated using one-way ANOVA followed by Tukey's multiple comparisons test. *, $P < 0.05$; **, $P < 0.01$; ***, $P < 0.001$; ****, $P < 0.0001$. ($n = 3$) (C) Dose response curve generated by using MDR505 mutant showed substantially higher neutralization. The error represents the s.d. of three replicates. All data was normalized to virus alone. (D) Calculated IC_{50} (mean ± s.d.) of each construct based on the SARS-CoV-2 pseudovirus neutralization.

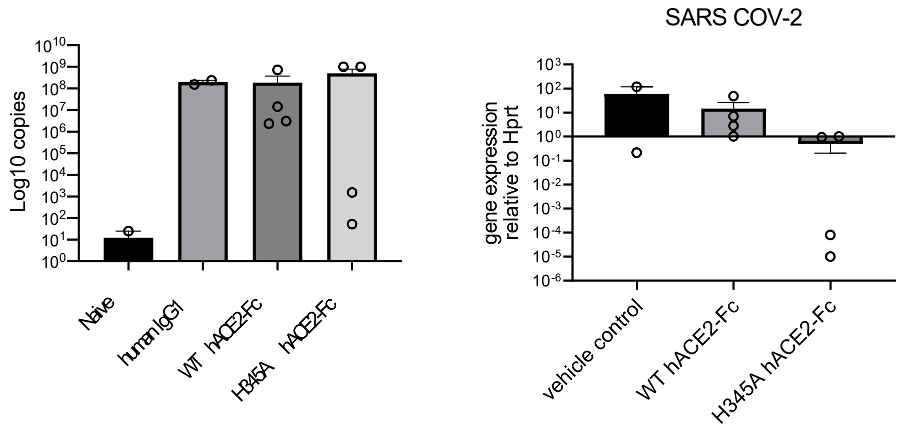

Figure S3. Subgenomic-N viral copies *In vivo* prophylaxis model. Related to Figure 4. Four days post oropharyngeal inoculation of Ad5-hACE2, 4 hours before SARS-CoV-2 challenge, mice were treated with 15 mg/ kg body weight human polyclonal IgG1, WT hACE2-Fc, or MDR504 hACE2-Fc intravenously. Three days later, mice were euthanized in ABSL3. SARS-CoV-2 infection was assayed by real time RT-PCR. Data are represented as mean +/- SEM. (*n* = 2-4, representative from 2 independent experiments).

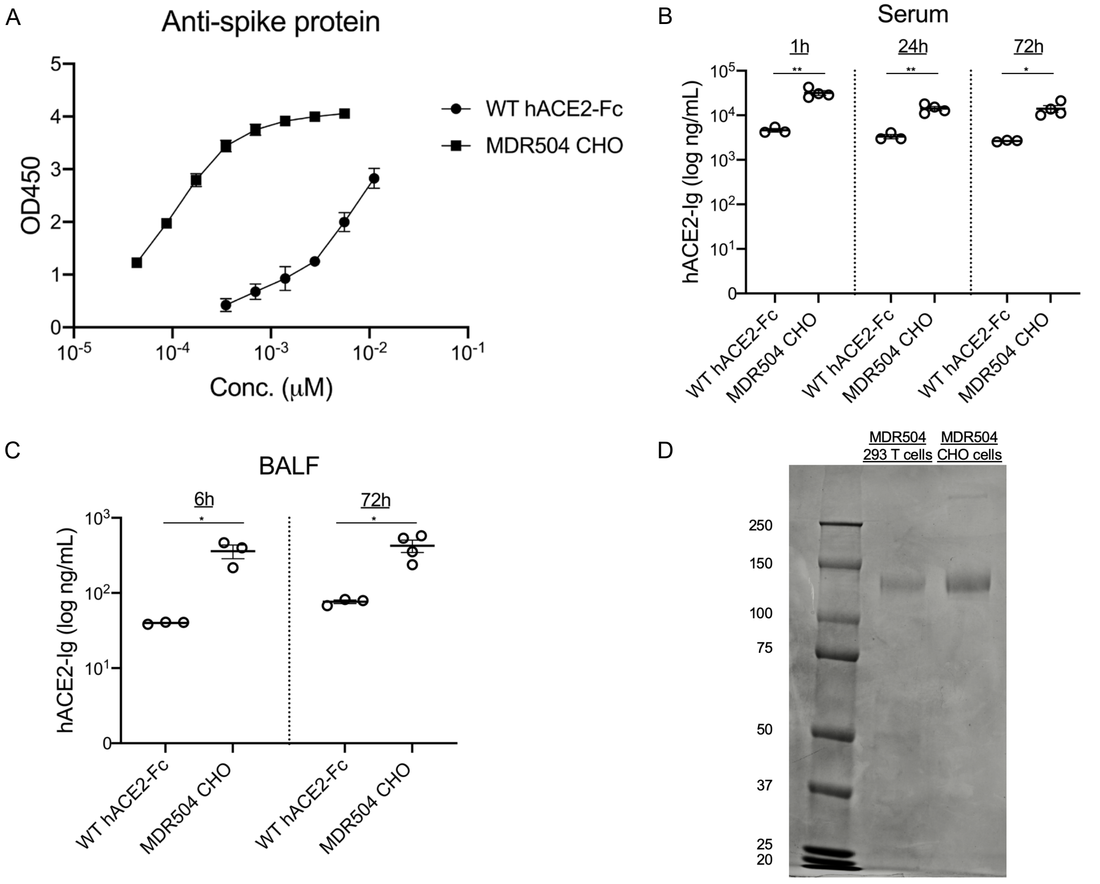

Figure S4. MDR504 generated from CHO cells. Related to Figure 4 and 5. (A) Binding of spike protein with MDR from CHO cells was compared to WT hACE2-Fc by ELISA. *In vivo* pharmacokinetics in serum (B) and BALF (C) of WT hACE2-IgG1 and MDR504 from CHO cells were assayed by ELISA post intravenous injection of 4 mg/ kg body weight of the protein. Data represent each value, the mean and SEM (n = 3-4). Significant differences are designated by using unpaired t-test. *, $P < 0.05$, **, $P < 0.01$ (*n* = 3-4, single experiment). (D) Western blotting under reducing condition with 2.5% 2-mercaptoethanol of MDR504 from 293T cells and CHO cells.

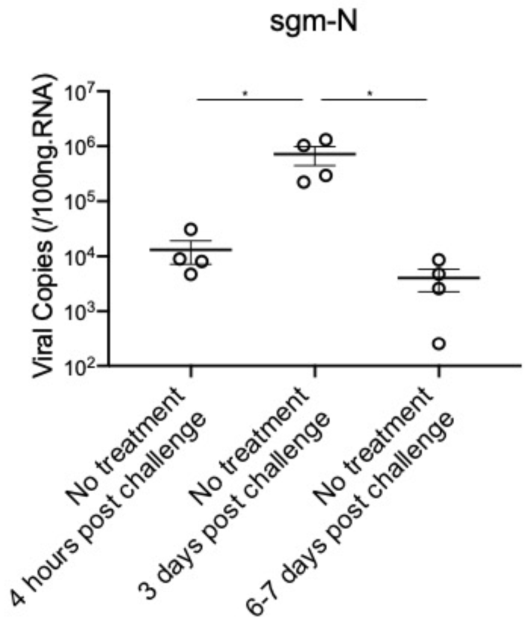

Figure S5. Subgenomic-N viral copies time course. Related to Figure 5. K18-hACE2 mice were infected with 2 x 10^5 TCID50 SARS-CoV-2, and subgenomic-N viral copies in lung tissue were determined at 4 hours, 3 days, and 6-7 days post challenge using real time RT-PCR. Data represent each value, the mean and SEM (n = 4). Significant differences are designated using one-way ANOVA followed by Tukey's multiple comparisons test. *, $P < 0.05$ (n = 4, cumulative data of two separate experiments).

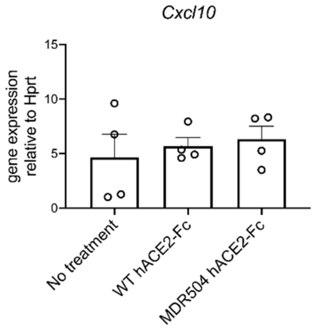

Figure S6. ***Cxcl10*** **gene expression** ***In vivo*** **treatment model. Related to Figure 5.** we administered 30 mg/kg body weight of MDR504 or control 4 hours after viral challenge. 72-hour s post challenge, mice were euthanized, and lung *Cxcl10* gene expression was measured by real time RT-PCR. Data represent each value, the mean and SEM (n = 4).

Table S1. CoVIC data shows a better neutralization of MDR504, related to Figure 2.

Inhibitor	CoVIC ID	Epitope Community (RBD)	Affinity, KD(M)			Pseudovirus neutralization (ng/mL)			Authentic virus neutralization (ng/mL)	
			D614	D614G	RBD	IC50	IC80	IC90	IC50	IC80
WT hACE2-Fc	69	1	1.82E -09	3.14E -10	2.67E -08	6840	20200	35400	482	2470
MDR504	89	1	1.62E -09	8.03E -10	6.73E -08	536	2130	4470	417	1117

Table S2. SARS-CoV2 spike variants, Related to STAR methods.

R & D	Cat #
Recombinant SARS-CoV-2 G476S Spike RBD His-tag Protein, CF	10627-CV-100
Recombinant SARS-CoV-2 V483A Spike RBD His-tag Protein, CF	10628-CV-100
Recombinant SARS-CoV-2 Spike RBD His-tag Protein, CF	10500-CV-100
**Acro Biosystem**	**Cat #**
SARS-CoV-2 (COVID-19) S protein RBD (E484K), His Tag (MALS verified)	SRD-C52H3-100
SARS-CoV-2 (COVID-19) S protein RBD (N439K), His Tag (MALS verified)	SRD-C52Hg-100
SARS-CoV-2 (COVID-19) S protein RBD (Y453F), His Tag (MALS verified)	SRD-C52Hk-100
SARS-CoV-2 (COVID-19) S protein (HV69-70del, N501Y, D614G), His Tag	S1N-C52Hk-100
SARS-CoV-2 (COVID-19) S protein RBD, His Tag	SPD-C52H1-200
**BEI Resources**	**Cat #**
Spike Glycoprotein (Stabilized) from SARS-Related Coronavirus 2, Wuhan-Hu-1 with C-Terminal Histidine Tag	NR-52397
Spike Glycoprotein (Stabilized) from SARS-Related Coronavirus 2, Delta Variant with C-Terminal Histidine and Avi Tags	NR-55614

Table S3. Antibodies for immunohistochemistry, related to STAR Methods.

**Primary Antibody**	**Vendor**	**Isotype**	**Dilution**	**Cat #**
Polyclonal Anti-SARS Coronavirus	BEI	Guinea Pig Rabbit	1:1000	NR-10361
Anti-CD31	R&D	Goat	1:100	AF-3628
Anti-VCAM-1	Abcam	Rabbit	1:250	Ab134047
**Secondary Antibody**	**Vendor**	**Fluorochrome**	**Dilution**	**Cat #**
Goat anti-Guinea Pig	Invitrogen	Alexa Fluor 488	1:1000	A-11073
Donkey anti-Goat	Invitrogen	Alexa Fluor 488	1:1000	A-11055
Donkey anti-Rabbit	Invitrogen	Alexa Fluor 647	1:1000	A-31573
